# Sulfoxide-Containing Bisabolane Sesquiterpenoids with Antimicrobial and Nematicidal Activities from the Marine-Derived Fungus *Aspergillus sydowii* LW09

**DOI:** 10.3390/jof9030347

**Published:** 2023-03-12

**Authors:** Xiao Yang, Hongjia Yu, Jinwei Ren, Lei Cai, Lijian Xu, Ling Liu

**Affiliations:** 1 State Key Laboratory of Mycology, Institute of Microbiology, Chinese Academy of Sciences, Beijing 100101, China; 2 College of Agricultural Resource and Environment, Heilongjiang University, Harbin 150080, China; 3 University of Chinese Academy of Sciences, Beijing 100039, China

**Keywords:** marine-derived fungi, *Aspergillus sydowii*, secondary metabolites, antibacterial activity, antifungal activity, nematicidal activity

## Abstract

Phytopathogens, such as phytopathogenic bacteria, fungi, and nematodes, have caused great losses of crops every year, seriously threatening human health and agricultural production. Moreover, marine-derived fungi are abundant sources of structurally unique and bioactive secondary metabolites that could be potential candidates for anti-phytopathogenic drugs. One new sulfoxide-containing bisabolane sesquiterpenoid aspersydosulfoxide A (**1**) and nine known analogues (**2**–**10**) were isolated from the marine-derived *A. sydowii* LW09. The absolute configuration of the sulfur stereogenic center in **1** was determined by electronic circular dichroism (ECD) calculations. Compound **5** showed inhibition activity against *Pseudomonas syringae*, with a minimum inhibitory concentration (MIC) value of 32 μg/mL, whereas, compounds **2**, **7**, and **8** showed antibacterial activities toward *Ralstonia solanacarum*, with the same MIC value at 32 μg/mL. Meanwhile, compounds **3**, **7**, and **8** inhibited the fungal spore germination of *Fusarium oxysporum*, with the half maximal effective concentration (EC_50_) values of 54.55, 77.16, and 1.85 μg/mL, respectively, while compounds **2**, **3**, **7**, and **8** inhibited the fungal spore germination of *Alternaria alternata*, which could be induced by vacuolization of germ tubes, with EC_50_ values of 34.04, 44.44, 26.02, and 46.15 μg/mL, respectively. In addition, compounds **3**, **7**, and **8** exhibited nematicidal activities against *Meloidogyne incognita* second-stage juveniles (J2s). In addition, compound **8** possessed the strongest nematicidal activity of nearly 80% mortality at 60 h with the half lethal concentration (LC_50_) values of 192.40 μg/mL. Furthermore, compounds **3**, **7**, and **8** could paralyze the nematodes and then impair their pathogenicity.

## 1. Introduction

Phytopathogens have caused great losses of crops every year, seriously threatening human health and agroindustry [1]. As the major part of phytopathogens, bacteria, fungi, and nematodes can infect many important economic crops, such as potato, soybean, wheat, and rice [2], leading to significant economic and production losses. Although traditional synthetic drugs are quite effective in managing pathogens, the residue accumulation and chemical resistance problems severely polluted the ecosystem and decreased the drug potency [3,4]. Therefore, searching for alternative molecules for anti-phytopathogenic drugs is urgent.

Fungi have been identified as a prolific source of secondary metabolites with effective properties of applications in pharmacy, food, and agriculture [5]. Specifically, in agronomy, many secondary metabolites exhibited significant antimicrobial activities against phytopathogens [6]. As clear examples, bioactive secondary metabolites produced by *Metarhizium*, *Beauveria*, and *Trichoderma* spp. have great potential in controlling phytopathogenic fungi, bacteria, and pests [7,8]. Marine-derived fungi, inhabiting special habitats such as high salinity, high pressure, absence of sunlight, and deficiency of nutrients, have been proved to be abundant sources of structurally unique and bioactive secondary metabolites for countering the biotic and abiotic stress [9,10]. The genus *Aspergillus* is prolific and ubiquitous in marine habitats, some of them could produce a variety of secondary metabolites with different structures, including polyketides, alkaloids, sterols, terpenoids, and peptides [11]. Many of these metabolites showed a wide range of bioactivities, such as antimicrobial, cytotoxic, insecticidal, and antioxidant activities [12]. During our ongoing efforts to search for new bioactive compounds from marine-derived fungi [13,14,15,16], a strain of *A*. *sydowii* LW09 isolated from a deep-sea sediment of the Southwest Indian Ridge was screened out for investigations. The EtOAc crude extract from the fermentation of this fungus showed antibacterial and antifungal activities. Bioassay-guided fractionation of this extract was performed, leading to the isolation of one new sulfoxide-containing phenolic bisabolane sesquiterpenoid aspersydosulfoxide A (**1**) and nine known analogues aspergillusene B (**2**), (−)-(*R*)-cyclo-hydroxysydonic acid (**3**), penicibisabolane G (**4**), (7*S*,11*S*)-(+)-12-hydroxysydonic acid (**5**), 11,12-dihydroxysydonic acid (**6**), expansol G (**7**), (*S*)-sydonic acid (**8**), aspergoterpenin C (**9**), and aspergillusene A (**10**). All the isolated compounds were tested for antibacterial activities against *P*. *syringae* and *R*. *solanacarum*, spore germination inhibition of *F*. *oxysporum* and *A*. *alternata*, and nematicidal activities against *M*. *incognita* J2s. Details of the isolation, structural elucidation, and bioactivities of these compounds are described herein.

## 2. Materials and Methods

### 2.1. Molecular Identification

Fungal genomic DNA of the strain LW09 was extracted from mycelia on a potato dextrose agar medium (PDA) using a previously published method [17]. For molecular analysis, the internal transcribed spacer (ITS), beta-tubulin (BenA), and calmodulin (CaM) regions were amplified using primer pairs ITS1 and ITS4, Bt2a and Bt2b, and cmd5 and cmd6, respectively [17]. PCR reactions were prepared following the published method [17]. Sequencing reactions were performed by Tsingke Biotechnology Co., Ltd., Beijing, China. All the sequences generated in this study were deposited in GenBank (ITS OP250138.1, BenA OP584347, and CaM OP584348). To determine the phylogenetic relationships of LW09, analysis was performed based on the three loci. Alignments were generated and manually edited by MEGA X (MUSCLE). Alignments of each locus were concatenated and used in the subsequent phylogenetic analysis. Maximum likelihood (ML) analysis was performed using RAxML v.7.4.2 Black Box (the code was released by Alexandros Stamatakis and Wayne Pfeiffer, US) in the CIPRES Science Gateway platform (https://www.phylo.org, accessed on 25 September 2022) with 1000 bootstrap replicates; *Aspergillus aurantiobrunneus* NRRL 4545 was used as an outgroup (Table S1).

### 2.2. General Experimental Procedure

IR spectra were recorded using a Nicolet IS5 FT-IR spectrophotometer (Thermo Scientific, Madison, WI, USA). UV/vis spectra were recorded using a Thermo Scientific Genesys 10S spectrophotometer (Thermo Scientific, Madison, WI, USA). Optical rotations were obtained by an Anton Paar MCP 200 Automatic Polarimeter (Anton Paar GmbH, Graz, Austria). ECD spectra were obtained with an Applied Photophysics Chirascan spectropolarimeter (Applied Photophysics Ltd., Leatherhead, UK). The NMR data were recorded with a Bruker Avance-500 MHz spectrometer (Bruker, Rheinstetten, Germany) using solvent signals (Acetone-*d*_6_: *δ*_H_ 2.05/*δ*_C_ 29.8, 206.1) as references. The mass data were acquired with an Agilent Accurate-Mass-Q-TOF LC/MS 6520 instrument (Agilent Technologies, Santa Clara, CA, USA). PCR reactions were conducted using a T20D PCR instrument (LongGene, Hangzhou, China). Column chromatography (CC) was accomplished on silica gel (Qingdao Haiyang Chemical Co., Ltd., Qingdao, China), octadecylsilyl (ODS, 50 μm, YMC Co., Ltd., Kyoto, Japan), and Sephadex LH-20 (Amersham Biosciences, Uppsala, Sweden). Preparative HPLC was conducted with an Agilent 1200 HPLC using a C18 column (Reprosil-Pur Basic C18 column; 5 μm; 10 × 250 mm) at a flow rate of 2.0 mL/min. The absorbance of compounds in the 96-well plates was detected by a SpectraMax Paradigm microplate reader (Molecular Devices, Sunnyvale, CA, USA). Aureomycin (Coolaber Science & Technology Co., Ltd., Beijing, China), chlorothalonil (Shanghai yuanye Bio-Technology Co., Ltd., Shanghai, China), ivermectin (Shanghai yuanye Bio-Technology Co., Ltd., Shanghai, China), and Abamectin (Shanghai yuanye Bio-Technology Co., Ltd., Shanghai, China) were used as positive controls.

### 2.3. Fungal Materials, Cultivation, Fermentation, and Isolation

The fungus sample *A*. *sydowii* LW09, deposited in the Institute of Microbiology, Chinese Academy of Sciences, Beijing, was isolated from a deep-sea sediment of the Southwest Indian Ridge (37°48′36” S; 49°39′36” E) at a depth of 2395 m. The strain was cultured on a PDA medium at 28 °C for 5 d. Afterward, four 0.5 cm^3^ plugs of agar with mycelia were inoculated in a 500 mL Erlenmeyer flask containing 250 mL liquid medium (0.4% glucose, 1% malt extract, and 0.4% yeast extract), then cultivated at 28 °C for 5 d on a rotary shaker at 200 rpm; subsequently, seed liquid culture was obtained and 5–10 mL spore suspension was transferred directly into a 500 mL Erlenmeyer flask with rice medium (80 g rice and 120 mL water) and fermented at 28 °C for 30 d in the dark. Afterward, a total of 10 kg fermentation sample was extracted three times with EtOAc (3 × 12 L); then, the filtrated organic solvent was evaporated in vacuo to obtain dryness extract (70.0 g). The dryness crude extract was fractionated by silica gel CC eluted with a gradient of petroleum ether (PE)/EtOAc (from 20:1 to 1:2, *v*/*v*) to give 10 fractions (Fr.1–Fr.10). The Fr.8 (0.6 g, eluted with PE/EtOAc 2:1) was repeatedly chromatographed by octadecylsilyl column chromatography (ODS CC), eluting with MeOH/H_2_O to yield four subfractions (Fr.8.1–Fr.8.4). The subfraction Fr.8.1 (48.0 mg, eluted with 50% MeOH) was further purified by RP-HPLC (Agilent Zorbax SB-C18 column; 5 μm; 10 × 250 mm; 60% CH_3_CN/H_2_O for 23.0 min; 2.0 mL/min) to give compounds **1** (2.5 mg, *t*_R_ = 17.0 min) and **5** (2.2 mg, *t*_R_ = 19.7 min). The Fr.4 (0.6 g, eluted with PE/EtOAc 8:1) was separated by ODS column eluting with MeOH/H_2_O and obtained five subfractions (Fr.4.1–Fr.4.5). The Fr.4.2 (42.3 mg, eluted with 60% MeOH) was purified by RP-HPLC (40% CH_3_CN/H_2_O for 32.0 min; 2.0 mL/min) to yield compounds **2** (3.0 mg, *t*_R_ = 30.0 min) and **9** (3.0 mg, *t*_R_ = 21.1 min). Compounds **3** (1.5 mg, *t*_R_ = 10.8 min) and **7** (1.7 mg, *t*_R_ = 19.0 min) were isolated by RP-HPLC (35% CH_3_CN/H_2_O for 20.0 min; 2.0 mL/min) from the Fr.4.5 (35.0 mg, eluted with 100% MeOH). The Fr.8.4 (55.0 mg, eluted with 100% MeOH) was further purified by RP-HPLC (67% MeOH/H_2_O for 35.0 min; 2.0 mL/min) to yield compounds **4** (2.4 mg, *t*_R_ = 30.0 min), **6** (3.0 mg, *t*_R_ = 24.5 min), and **8** (1.9 mg, *t*_R_ = 23.0 min). The Fr.3 (0.9 g, eluted with PE/EtOAc 12:1) was separated by ODS column eluting with MeOH/H_2_O, yielding three fractions (Fr.3.1–Fr.3.3). The Fr.3.2 (0.3 g, eluted with 80% MeOH) was further purified by RP-HPLC (73% MeOH/H_2_O for 21.0 min; 2.0 mL/min) to yield compound **10** (2.8 mg, *t*_R_ = 19.0 min). The flowchart of the compounds is given in Figure S11.

Aspersydosulfoxide A (**1**): colorless oil, ${\left[\alpha \right]}_{D}^{25}$ = −2.0 (*c* 0.05, MeOH); UV (MeOH) *λ*_max_ (log *ε*) 228 (3.70), 244 (3.66), 288 (3.32) nm; IR (MeOH) *ν*_max_ 3178, 2954, 2926, 1643, 1610, 1577, 1466, 1424, 1294, 1167, 1036, 1024, 979, 817 cm^−1^; ECD (0.36 mM, MeOH) *λ*_max_ (Δ*ε*) 230 (+1.25) nm, 253 (+0.38) nm; HRESIMS *m*/*z* 281.1571 [M+H]^+^ (calcd for C_16_H_25_O_2_S, 281.1575). For ^1^H and ^13^C NMR data, see Table 1.

### 2.4. ECD Calculation Methods

Conformational analysis within an energy window of 3.0 kJ/mol was performed by using the OPLS3 molecular mechanics force field. The conformers were then further optimized with the software package Gaussian 09 at the B3LYP/6-311G (d,p) level, and the harmonic vibrational frequencies were also calculated to confirm their stability. Then, the 60 lowest electronic transitions for the obtained conformers in vacuum were calculated using time-dependent density functional theory (TDDFT) methods at the CAM-B3LYP/6-311G (d,p) level. ECD spectra of the conformers were simulated using a Gaussian function. The overall theoretical ECD spectra were obtained according to the Boltzmann weighting of each conformer [18].

### 2.5. The Antibacterial Assay

The antibacterial assay was performed according to the previous method [19]. Two typical phytopathogens (*P*. *syringae* BLY016 and *R*. *solanacarum* BLY014) were cultured in LB broth (1% peptone, 0.5% yeast extract, and 1% NaCl) at 38 °C for 24 h under static condition, and then the concentration of bacterial cells was adjusted to approximately 1 × 10^6^ CFU/mL and 100 μL aliquots were inoculated in 96-well microtiter plates containing 100 μL of the isolated compounds diluted serially two-fold. The concentrations of tested compounds were ranged from 0.5 to 64 μg/mL. After incubation at 38 °C for 24 h, MIC was determined by microplate spectrophotometer. All experiments were performed in triplicate.

### 2.6. The Inhibition of Spore Germination Assay

The inhibition of pathogen spore germination assay was performed according to the previous study [20] with some modifications. The fungal pathogens (*F*. *oxysporum* FLY001 and *A*. *alternata* LW37) were cultivated on PDA media at 28 °C for 7 d. The 0.5% sterilized glucose buffer was used to rinse pathogen culture media and then filtrated through a syringe with four layers of sterilized gauze to yield spore suspensions. The working solution of the suspensions was adjusted to approximately 1 × 10^5^–1 × 10^6^ spores/mL. Spore suspensions of 100 μL were inoculated in 96-well microtiter plates containing 100 μL of the isolated compounds diluted serially two-fold, and the ranges were from 1 to 128 μg/mL. After cultivation at 28 °C for 24 h, 60 spores were observed by microscope and the germinated ones were counted. The spore germination rate was calculated with the formula given below. DMSO and chlorothalonil were used as negative and positive controls. All experiments were operated in triplicate and the data were presented as mean ± SD of three replicates. Afterwards, the EC_50_ values were generated by GraphPad prism 7.0 with different concentrations of the tested compounds and their spore germination rates.

(1) $$\mathrm{Spore}\;\mathrm{germination}\;\mathrm{rate}\;(\%)=\frac{\mathrm{Treated}\;\mathrm{germinated}\;-\;\mathrm{positive}\;\mathrm{germinated}}{\mathrm{Total}\;\mathrm{observed}\;\mathrm{spores}}\;\times \;100\%$$

### 2.7. The Nematode Toxic Assay

The nematode toxic assay was designed following the method [21] with some modifications. Eggs of *M. incongnita* were collected from the root of *Ipomoea aquatica* Forsk pot cultures. Afterward, the *M. incongnita* J2s hatched in the dark in sterile water for 24 h at 28 °C. Newly emerged J2s were then washed three times in sterile water before being used in the assays. To evaluate the toxicity of the compounds, J2s were diluted to about 1000 individuals/mL and the stock solution of the compounds was prepared at 10 mg/mL for further dilution to the required concentrations. Firstly, all the compounds were screened for their nematicidal activities against J2s at 800 μg/mL with a 48 h duration. Then, the active ones were set to 50, 100, 200, and 400 μg/mL in 96-well plates and incubated under 28 °C, with the death number of J2s recorded at set intervals. Before counting, 0.5 M NaOH was added to wells, which allowed the dead and alive nematodes to be clearly distinguished, as the living ones huddled when contacted and straight and immobile ones were defined as dead; then, the corrected mortalities were calculated by the formula given below. The mobility observations were determined by the wiggly frequency of nematodes; motionless ones were recognized as “−”, under 5 times per 10 s were identified as “+”, and upper ones were “++”. DMSO was used as a negative control; abamectin and ivermectin were used as positive control. The experiment was performed with three replicates and the LC_50_ values were calculated by GraphPad prism 7.0 with different concentrations of the tested compounds and the corrected mortality.

(2) $$\mathrm{The}\;\mathrm{corrected}\;\mathrm{mortality}\;(\%)=\frac{\left(\mathrm{Treated}\;\mathrm{mortality}\;-\;\mathrm{Control}\;\mathrm{mortality}\right)}{\left(1\;-\;\mathrm{Control}\;\mathrm{mortality}\right)}\;\times \;100\%$$

### 2.8. Data Analysis and Process

The NMR data analysis used MestReNova 14. The sequences were processed by MEGA X, BLAST (https://blast.ncbi.nlm.nih.gov/Blast.cgi, accessed on 23 September 2022), and CIPRES (https://www.phylo.org, accessed on 26 February 2023) for identifying the isolated fungus. The corrected mortalities of the compounds were compared by the Tukey test with Origin 2021. All analyses and plots were conducted using Origin 2021 (Northampton, MA, USA), GraphPad prism 7.0 (San Diego, CA, USA), Adobe Illustrator 2021 (San Jose, CA, USA), and Office Excel 2016 (Redmond, DC, USA).

## 3. Results

### 3.1. Phylogenetic Analysis

**Figure 1:**
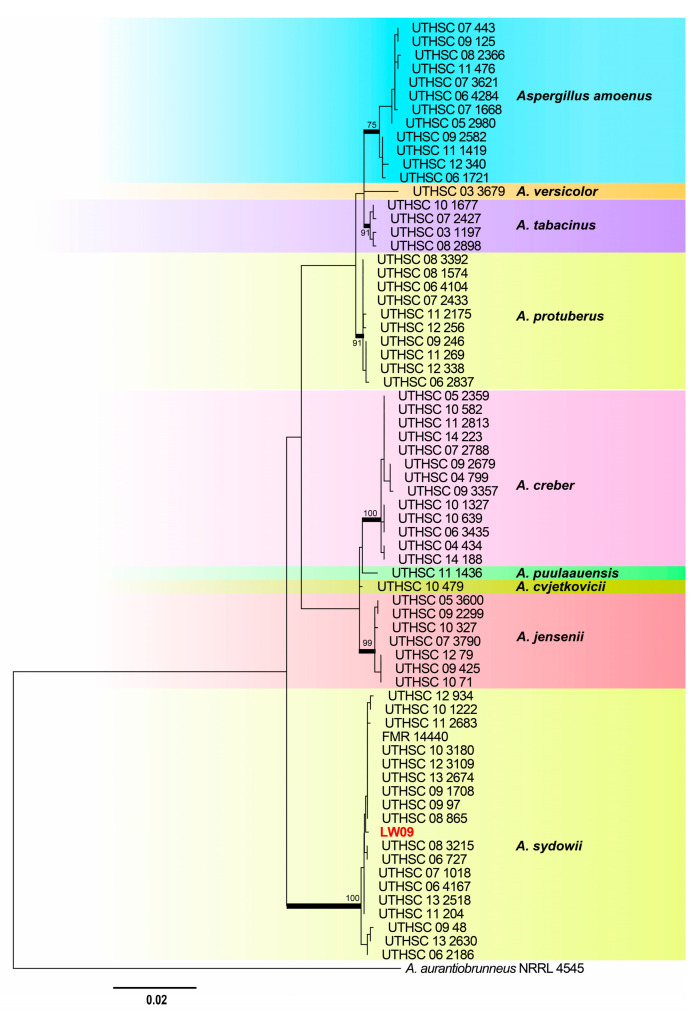
Maximum likelihood analysis of several *Aspergillus* species based on ITS, BenA, and CaM sequences. Bootstrap values ≥75% were indicated at the nodes. The tree was rooted to *A. aurantiobrunneus* NRRL 4545.

The phylogenetic analysis based on three loci (ITS, BenA, and CaM) was constructed using ML analysis (Figure 1). Phylogenetically, the strain LW09 was clustered in the known strains of *A. sydowii* (Bootstrap values = 100%). Thus, the strain LW09 was identified as *A. sydowii*.

### 3.2. Structure Elucidation

**Figure 2:**
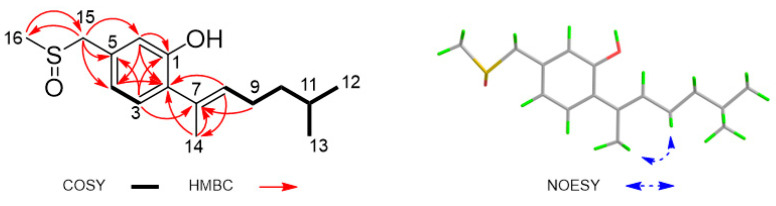
Key ^1^H-^1^H COSY, HMBC, and NOESY correlations of compound **1**.

Aspersydosulfoxide A (**1**) was initially obtained as deep yellow oil. Its molecular formula was determined as C_16_H_24_O_2_S by HRESIMS (*m*/*z* 281.1571 [M + H]^+^, calcd for C_16_H_25_O_2_S 281.1575), indicating five degrees of unsaturation. The IR spectrum indicated the presence of a hydroxy group (3178 cm^−1^), an aromatic ring (1643, 1610, and 1577 cm^−1^) and a sulfoxide functional group (1036 cm^−1^) (Figure S2). The ^1^H NMR spectrum (Figure S3) of **1** exhibited resonances for three aromatic protons at *δ*_H_ 7.05 (d, *J* = 7.7 Hz, H-3), 6.84 (s, H-6), and 6.77 (dd, *J* = 7.7 Hz, 1.4 Hz, H-4), an olefinic proton at *δ*_H_ 5.46 (tq, *J* = 7.4 Hz, 1.2 Hz, H-8), one sp^3^ methine proton at *δ*_H_ 1.63 (m, H-11), six methylene protons at *δ*_H_ 3.94 (d, *J* = 13.0 Hz, H-15a), 3.85 (d, *J* = 13.0 Hz, H-15b), 2.18 (q, *J* = 7.4 Hz, H_2_-9), and 1.33 (m, H_2_-10), and four methyls at *δ*_H_ 2.46 (s, H_3_-16), 1.99 (s, H_3_-14), 0.92 (d, *J* = 6.5 Hz, H_3_-13), and 0.92 (d, *J* = 6.5 Hz, H_3_-12). The ^13^C NMR and HSQC data (Figures S4 and S5) of **1** showed 16 carbon signals, including four methyls (*δ*_C_ 38.1, 22.9, 22.9, and 17.1), three sp^3^ methylenes (*δ*_C_ 60.3, 39.5, and 26.9), one sp^3^ methine (*δ*_C_ 28.4), four sp^2^ methines (*δ*_C_ 130.9, 130.3, 122.2, and 118.1), and four quaternary carbons (*δ*_C_ 155.0, 134.7, 133.3, and 131.7) (Table 1). These data accounted for all ^1^H and ^13^C NMR resonances of **1**, except for one unobserved hydroxyl group, one oxygen atom, and one sulfur atom. In the HMBC spectrum (Figure S7), the correlations from the aromatic protons H-6 to C-1, C-2, C-4, and C-15, from H-4 to C-2, C-3, C-5, C-6, and C-15, from H-3 to C-1, C-2, C-4, C-5, and C-7, and from the methylene protons H_2_-15 to C-4, C-5, and C-6 together with the ^1^H-^1^H COSY correlations (Figure S6) of H-3/H-4 (Figure 2) constructed the 1,2,5-trisubstituted benzene ring with the methylene carbon C-15 substituted at C-5. Other HMBC correlations from H-3 and H-9 to the quaternary carbon C-7 (*δ*_C_ 134.7), H-8 to C-2 and C-14, and from H_3_-14 to C-2, C-7, and C-8, combined with ^1^H-^1^H COSY correlations of H-8/H_2_-9/H_2_-10/H-11/H_3_-12/H_3_-13, indicated the presence of methylhept-2-en-2-yl group located at C-2 position of the benzene ring. The hydroxyl group was located at C-1 by default, which was supported by the chemical shift of C-1 (*δ*_C_ 155.0). The chemical shifts of the methyl group CH_3_-16 (*δ*_H/C_ 2.46/38.1) and the methylene group CH_2_-15 (*δ*_H/C_ 3.94, 3.85/60.3) indicated that both carbons were attached to a hetero atom. Considering the molecular formula of **1**, the sulfinyl group deriving from the remaining one oxygen atom and one sulfur atom should be inserted between two carbons, C-15 and C-16, to form a methylsulfinyl substituent, which was further confirmed by the key HMBC correlations from H_2_-15 to C-16 and from H_3_-16 to C-15. Thus, the planar structure of **1** was established as shown (Figure 2).

**Figure 3:**
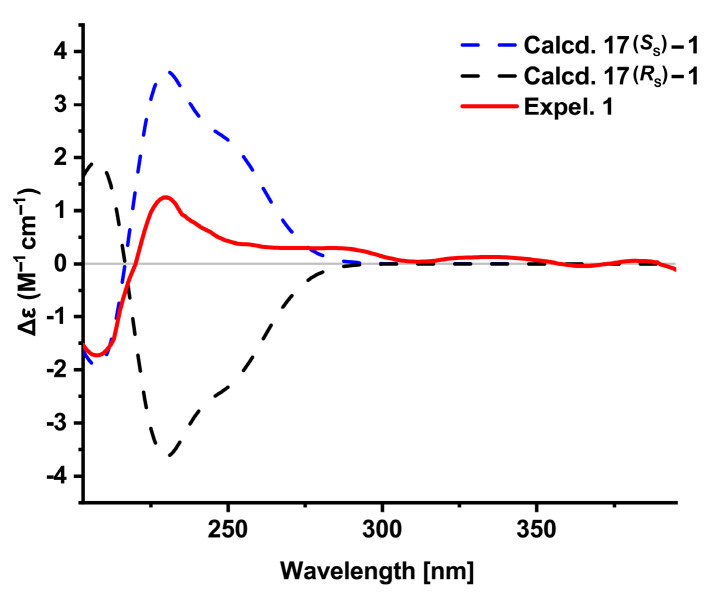
Experimental ECD spectrum of **1** and the calculated ECD spectra of (*S*_S_)-**1** and (*R*_S_)-**1**.

The *E* geometry of the olefin C-7/C-8 was assigned by the NOESY correlation (Figure S8) of H_2_-9 with H_3_-14 (Figure 2). The absolute configuration of the sulfur stereogenic center in **1** was determined by comparison of the experimental ECD spectrum of **1** with those of the time-dependent density functional theory (TDDFT) calculations at the B3LYP/6-311G (d,p) level performed on (*S*_S_)-**1** and (*R*_S_)-**1** (Figure S9). As a result, the trend of the experimental ECD spectrum of **1** was identical to that of the calculated curve for (*S*_S_)-**1** (Figure 3), which indicated *S* configuration for the chiral sulfoxide of **1**.

**Figure 4:**
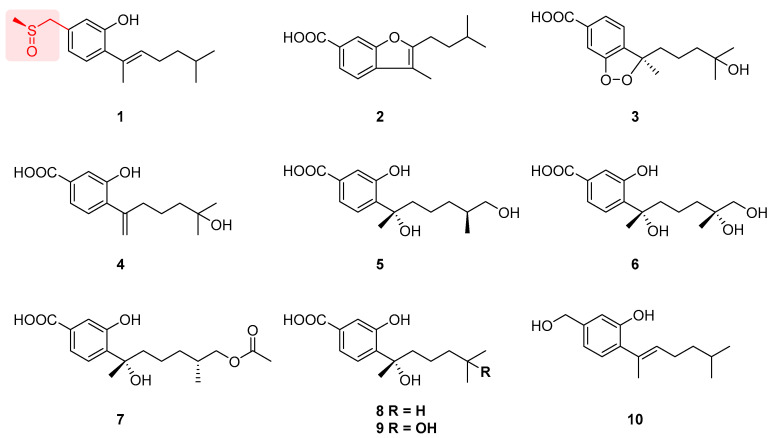
Structures of compounds **1**–**10**.

The known compounds were identified to be aspergillusene B (**2**) [22], (−)-(*R*)-cyclo-hydroxysydonic acid (**3**) [23], penicibisabolane G (**4**) [24], (7*S*,11*S*)-(+)-12-hydroxysydonic acid (**5**) [25], 11,12-dihydroxysydonic acid (**6**) [26], expansol G (**7**) [27], (*S*)-sydonic acid (**8**) [28], aspergoterpenin C (**9**) [29], and aspergillusene A (**10**) [22], respectively, by comparison with corresponding data in the literature (Figure 4).

### 3.3. Antibacterial Activities of the Isolated Compounds

Compounds **1**–**10** were evaluated for antibacterial activities against two phytopathogenic bacteria *P*. *syringae* and *R*. *solanacarum* using the broth microdilution method [30,31]. The MIC values of these compounds are shown in Table 2. Compound **5** showed modest antibacterial activity against *P*. *syringae*, with an MIC value of 32 μg/mL. Compounds **2**, **7**, and **8** showed modest antibacterial activity against *R*. *solanacarum*, with the same MIC value of 32 μg/mL.

### 3.4. Inhibition of Spore Germination of the Isolated Compounds

**Figure 5:**
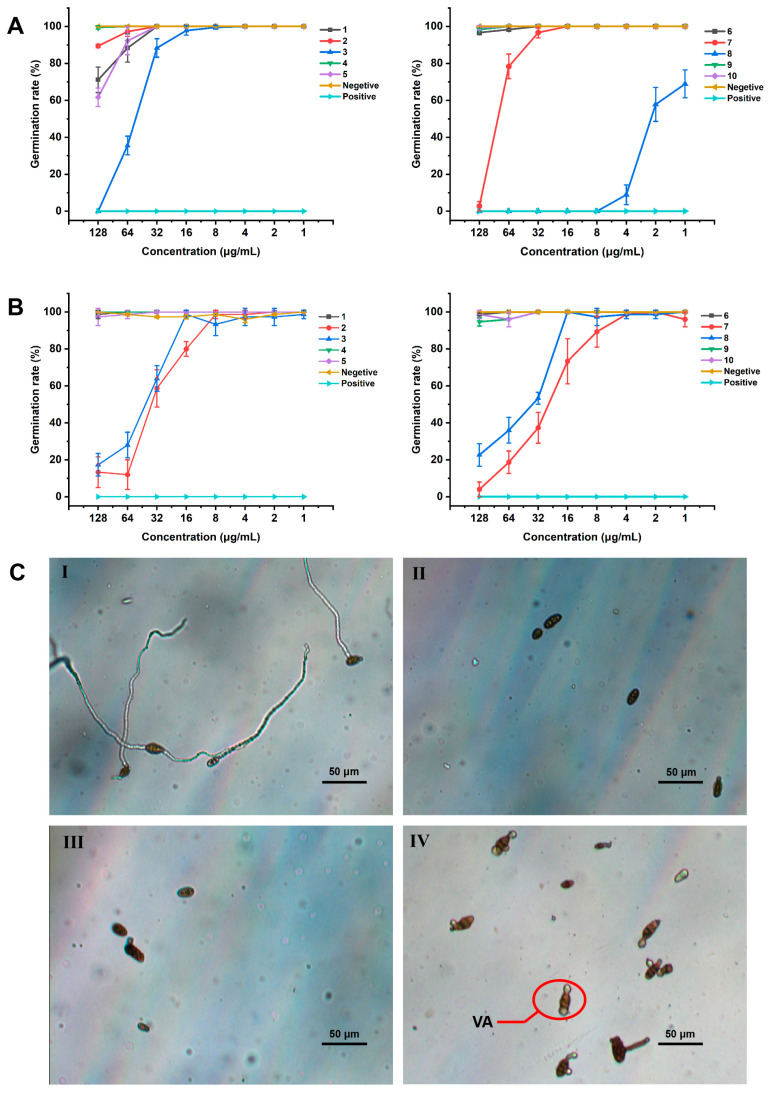
Germination rates of *F*. *oxysporum* (**A**) and *A*. *alternata* (**B**) treated with the tested compounds. Error bars indicate ± standard deviations, calculated from three replicates. DMSO was used as negative control and chlorothalonil was used as positive control. (**C**) Microscopy images of *A*. *alternata* spores with different treatments: (**I**) negative control at 128 μg/mL DMSO; (**II**) positive control at 1 μg/mL chlorothalonil; (**III**) compound **8** at 128 μg/mL; (**IV**) compound **8** at 32 μg/mL. VA means germ tube vacuolization.

Phytopathogenic fungi *F*. *oxysporum* and *A*. *alternata* are typical soil-borne and air-borne pathogens which caused huge losses of crops annually [32,33], and spore germination is an essential part of their disease cycle. The isolated compounds **1**–**10** were evaluated for their antifungal activities against the phytopathogenic fungi *F*. *oxysporum* and *A*. *alternata* using spore germination tests. Compounds **1**, **2**, **3**, **5**, **7**, and **8** could inhibit the spore germination of *F*. *oxysporum* in the concentration range of 128–32 μg/mL (Figure 5A). Compounds **3** and **7** inhibited the spore germination of *F*. *oxysporum*, with EC_50_ values 54.55 and 77.16 μg/mL, respectively (Table 3). Among the test compounds, compound **8** strongly inhibited the spore germination of *F*. *oxysporum*, with an EC_50_ value of 1.85 μg/mL (Table 3). Furthermore, compound **8** was not going into a plateau phase at 1 μg/mL according to the curve (Figure 5A), revealing that it could inhibit the spore germination of *F*. *oxysporum* at a lower concentration (<1 μg/mL). In addition, compounds **2**, **3**, **7**, and **8** also showed good inhibition against *A*. *alternata* spore germination in the range of 128–32 μg/mL (Figure 5B), with EC_50_ values of 34.04, 44.44, 26.02, and 46.15 μg/mL, respectively (Table 3). Further investigations showed that the spore germ tubes of *A*. *alternata* were vacuolated with the treatment of compounds **2**, **3**, **7**, and **8** (Figure 5C and Figure S10).

### 3.5. Nematicidal Activity of the Isolated Compounds

**Figure 6:**
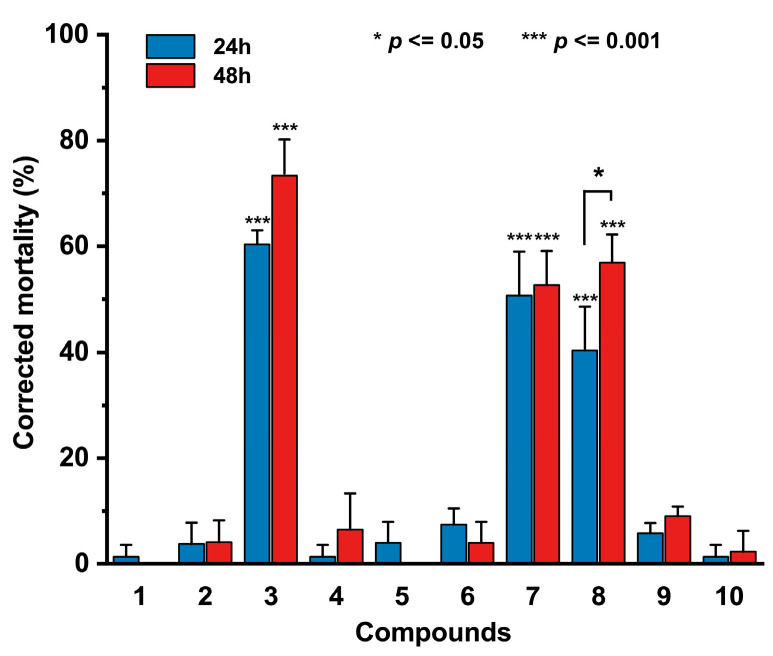
Corrected mortality of *M*. *incongnita* J2s treated with the tested compounds at 800 μg/mL. DMSO was used as negative control with the same concentration. Plotted are mean + standard deviations; each treatment set three replicates. Compounds **3**, **7**, and **8** represented statistically significant differences (the Tukey test, *p* ≤ 0.001) between active ones and inactive ones, and **8** represented significant differences between 24 h and 48 h treated procedures (the Tukey test, *p* ≤ 0.05).

**Figure 7:**
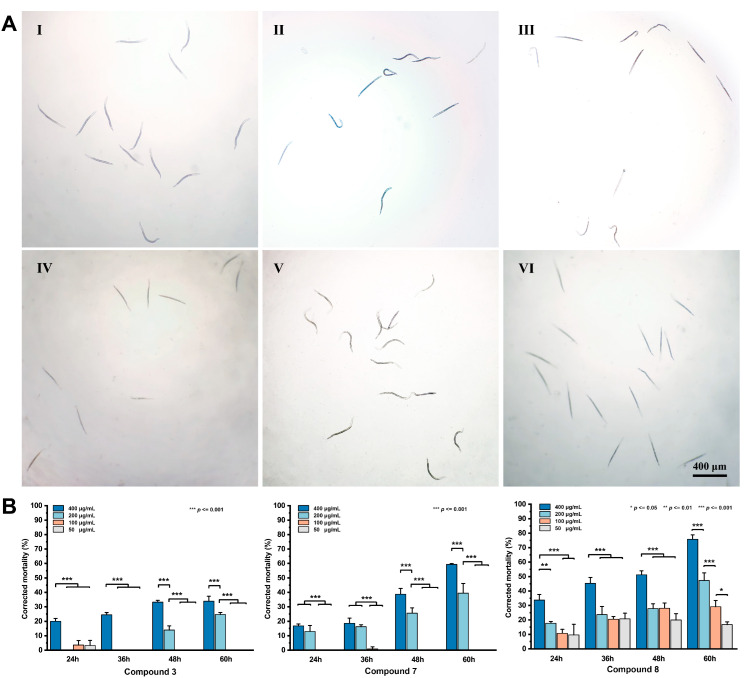
(**A**) Microscopy images of nematodes treated with 50–400 μg/mL compound **8** at 60 h (**I**–**IV**); (**V**) negative control at 400 μg/mL DMSO; (**VI**) positive control at 400 μg/mL abamectin. (**B**) Corrected mortality of *M*. *incongnita* treated with different concentrations of the tested compounds in different observation times. Graphs were presented with mean + standard deviations. Asterisk above the bars indicated significant differences (the Tukey test, * *p* ≤ 0.05; ** *p* ≤ 0.01; *** *p* ≤ 0.001).

The nematicidal activity of the isolated compounds was assessed against the soil nematode *M*. *incongnita* J2s. Compounds **1**–**10** were firstly evaluated for toxic effects against the *M*. *incongnita* J2s at 24 h and 48 h with 800 μg/mL. Compounds **3**, **7**, and **8** exhibited significant nematicidal activity, and compound **3** was the most effective one (Figure 6). Then, we tested the time–concentration dependency of *M*. *incongnita* treated with target compounds **3**, **7**, and **8**, which recorded the mortality at 12 h intervals from 24 to 60 h at the concentration range of 50–400 μg/mL (Figure 7B). Intriguingly, the nematicidal activity of compound **3** in these treatments was reduced, which might be caused by the treated concentration being close to its minimum lethal concentration. Additionally, the nematicidal activity of **8** became better than those of **3**, **7**, and ivermectin, with an LC_50_ value of 192.40 μg/mL, which was close to the abamectin positive control of 146.10 μg/mL at 60 h (Table 4). At the same time, nearly 80% of the *M*. *incongnita* J2s treated with 400 μg/mL of compound **8** were dead (Figure 7B), whereas compounds **3** and **7** showed modest toxic effects at 200–400 μg/mL with 20–60% corrected mortality, and the toxic effects at 400 μg/mL were significantly higher than those of 200 μg/mL (Figure 7B). These results revealed that the minimum lethal concentration of compound **8** was much lower than those of **3** and **7**, and the effective compounds exhibited time/concentration-dependent inhibition toward *M*. *incongnita* J2s.

Furthermore, we observed that living nematodes showed different mobility, as they kept wiggling or stayed still during the observation time treated with different concentrations of compounds. This phenomenon revealed that the target compounds **3**, **7**, and **8** might possess paralytic ability toward nematodes. Compounds **3** and **7** could paralyze nematodes at 400–200 μg/mL, while compound **8** could paralyze nematodes at 50 μg/mL. Additionally, the appearance of the reversible paralytic processes (Table 5) could be caused by the drug resistance of different observed individuals.

### 3.6. Plausible Biosynthetic Pathways of the Phenolic Bisabolane Sesquiterpenoids

**Scheme 1:**
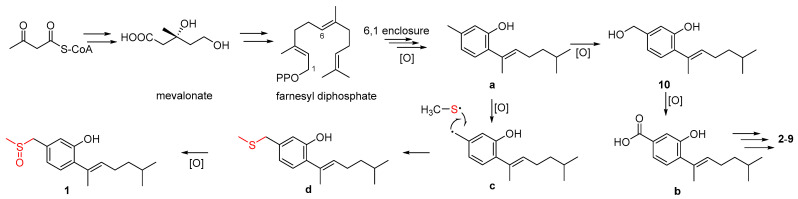
The plausible biosynthetic pathways of compounds **1**–**10**.

Biogenetically, the phenolic bisabolane skeleton (**a**) of compounds **1**–**10** could be derived from mevalonate-farnesyl diphophate-bisabolane pathway, as proposed in Scheme 1. The key intermediate **a** underwent an oxidation reaction to give **10**. Oxidation of C-15 of **10** afforded benzoic acid derivative **b**, which further underwent a series of oxidation, cyclization, reduction, and esterification to yield compounds **2**–**10**. In addition, the methylthio radical (CH_3_S•), possibly formed during the in vivo sulfur metabolism pathways, could be trapped by the methyl radical (H_3_C•), which could be generated by oxidation of benzyl of intermediate **a**, resulting in the production of methyl thioether **d**. The oxidation of methyl thioether **d** finally generated the methylsulfinyl bisabolane sesquiterpenoid **1**. 

## 4. Discussion

Fungal secondary metabolites have played a vital role in drug discovery [34]. With the tremendous chemical diversities and potent biological activities, some of the metabolites have great potential in agricultural applications, such as beauvericin and trichodermin [35,36]. Phenolic bisabolane sesquiterpenoids are a rare cluster of natural products, and most of them were obtained from marine fungi [26,27,29]. Structurally, phenolic bisabolane sesquiterpenoids were characterized by a *para*-alkylated benzene ring system and the structural variability of them was mainly caused by reduction, oxidation, and cyclization reactions of the side chain [37]. Meanwhile, the presence of the sulfoxide group was quite rare among phenolic bisabolane sesquiterpenoids, with only three compounds having been reported [37,38]. In this study, a rare sulfoxide containing phenolic bisabolane aspersydosulfoxide A (**1**) was isolated and identified. However, it did not show obvious activities in these bioassays. Compounds **2**, **5**, **7**, and **8** showed selective antibacterial activities against the phytopathogenic bacteria *P*. *syringae* and *R*. *solanacarum*, and the difference in antibacterial activities might be determined by their different functional groups on their side chains.

Spores are special forms in the fungal life cycle, and they possessed strong tolerance that can promote dissemination and keep long-term survival. Thus, compounds inhibiting spore germination could be developed into high-efficiency and low-toxicity drugs for preventing fungal diseases [39]. In our study, most of the phenolic bisabolane sesquiterpenoids inhibited the spore germination of *F*. *oxysporum* and *A*. *alternata*. Interestingly, the spore germ tubes of *A*. *alternata* were vacuolated with the treatment of compounds **2**, **3**, **7**, and **8**. Vacuolization could delay the spore germination progress of fungi [20]. Thus, we assumed that the phenolic bisabolane sesquiterpenoids **2**, **3**, **7**, and **8** could inhibit the spore germination procedure of *A*. *alternata* by vacuolization of germ tubes.

The root-knot nematode *M*. *incognita* is the main pest in tropical and subtropical regions that has caused great harm to many crops [40]. Second-stage juveniles are the most infective stage of *M*. *incognita*. They penetrated the root of the host and moved to the vascular cylinder through cortical tissue, then became sedentary [41]. Thus, controlling J2s might be an efficient management method for nematode disease. In previous studies, non-phenolic bisabolane sesquiterpenoids cheimonophyllons A–D and cheimonophyllal showed nematicidal activity [42], whereas the phenolic ones have not been investigated previously. In this study, the phenolic bisabolane sesquiterpenoids **3**, **7**, and **8** also exhibited nematicidal activity and limited the mobility of nematodes, indicating that the nematicidal activities of these sesquiterpenoids might depend on the groups of the aliphatic sidechain.

## 5. Conclusions

In summary, one new sulfoxide-containing phenolic bisabolane sesquiterpenoid aspersydosulfoxide A (**1**) and nine known congeners (**2**–**10**) were isolated from the marine-derived fungus *A*. *sydowii* LW09. The absolute configuration of the sulfur stereogenic center in **1** was determined by ECD calculations. The biosynthetic pathways of compounds **1**–**10** were proposed. Some of the isolated compounds showed selective antibacterial activities against the phytopathogenic bacteria *P*. *syringae* and *R*. *solanacarum*, and inhibited the spore germination of the phytopathogenic fungi *F*. *oxysporum* and *A*. *alternata*. Meanwhile, it is possible that compounds **2**, **3**, **7**, and **8** inhibited the spore germination procedure of *A*. *alternata* by vacuolization of germ tubes. The nematicidal activities of the phenolic bisabolane sesquiterpenoids **3**, **7**, and **8** were first reported. Our study not only expanded the chemical diversity of the phenolic bisabolane sesquiterpenoids, but also provided the potential lead compounds for anti-phytopathogenic drugs.

## Figures and Tables

**Table 1:** ^1^H (500 MHz) and ^13^C (125 MHz) NMR data of **1** in acetone-*d*_6_.

Position	*δ*_H_ *J* (Hz)	*δ*_C_, Type	HMBC
1		155.0, C	
2		133.3, C	
3	7.05, d (7.7)	130.3, CH	C-7, C-5, C-4, C-2, C-1
4	6.77, dd (7.7, 1.4)	122.2, CH	C-15, C-6, C-5, C-3, C-2
5		131.7, C	
6	6.84, s	118.1, CH	C-15, C-4, C-2, C-1
7		134.7, C	
8	5.46, tq (7.4, 1.2)	130.9, CH	C-14, C-10, C-9, C-2
9	2.18, q (7.4)	26.9, CH_2_	C-11, C-10, C-8, C-7
10	1.33, m	39.5, CH_2_	C-13, C-12, C-11, C-9, C-8
11	1.63, m	28.4, CH	C-13, C-12, C-10, C-9
12	0.92, d (6.5)	22.9, CH_3_	C-13, C-11, C-10
13	0.92, d (6.5)	22.9, CH_3_	C-12, C-11, C-10
14	1.99, s	17.1, CH_3_	C-8, C-7, C-2
15	3.85, d (13.0) 3.94, d (13.0)	60.3, CH_2_	C-16, C-6, C-5, C-4
16	2.46, s	38.1, CH_3_	C-15
1-OH	8.16, br s		

**Table 2:** Antibacterial activities of compounds **1**−**10**.

Organisms	MIC ^b^ Values (μg/mL)										
	1	2	3	4	5	6	7	8	9	10	Positive ^a^
*P. syringae*	>64	>64	>64	>64	32	>64	>64	>64	>64	>64	1
*R. solanacarum*	>64	32	>64	>64	>64	>64	32	32	>64	>64	0.125

^a^ Aureomycin was used as positive control. ^b^ Inhibition of bacterial growth reached 50%.

**Table 3:** Spore germination inhibition activities of compounds **1**−**10**.

Compounds	*F*. *oxysporum*	*A*. *alternata*
	EC_50_ (μg/mL) ^a^	
**1**	>128	>128
**2**	>128	34.04 ± 1.06
**3**	54.55 ± 1.02	44.44 ± 1.06
**4**	>128	>128
**5**	>128	>128
**6**	>128	>128
**7**	77.16 ± 1.02	26.02 ± 1.06
**8**	1.85 ± 1.07	46.15 ± 1.10
**9**	>128	>128
**10**	>128	>128
Chlorothalonil	<1	<1

^a^ The EC_50_ values were presented by mean ± standard deviation (calculated from three replicates by GraphPad prism 7.0).

**Table 4:** Nematicidal activities of **3**, **7**, and **8**.

Compounds	LC_50_ (μg/mL) ^a^			
	24 h	36 h	48 h	60 h
**3**	>400	>400	>400	>400
**7**	>400	>400	>400	302.8 ± 1.08
**8**	>400	>400	367.60 ± 1.06	192.40 ± 1.05
Ivermectin	>400	311.00 ± 1.21	273.80 ± 1.07	206.90 ± 1.07
Abamectin	375.40 ± 1.05	220.00 ± 1.05	192.80 ± 1.04	146.10 ± 1.07

^a^ The LC_50_ values were presented by mean ± standard deviation (calculated from three replicates by GraphPad prism 7.0).

**Table 5:** The mobility of *M*. *incongnita*.

Observation Time (h)	Treated Concentration (μg/mL)												
	3				7				8				*p*
	400	200	100	50	400	200	100	50	400	200	100	50	50
24	–	+	++	++	–	–	+	+	–	–	–	–	–
36	–	++	++	++	–	–	++	++	–	+	+	+	–
48	–	++	++	++	–	+	++	++	–	+	+	+	–
60	–	+	++	++	–	–	+	++	–	–	+	+	–

## Data Availability

All data generated or analyzed in this study are available within the manuscript and are available from the corresponding authors upon request.

## Supplementary Materials

The following supporting information can be downloaded at: https://www.mdpi.com/article/10.3390/jof9030347/s1, Figure S1: HRESIMS spectrum of compound **1**, Figure S2: IR spectrum of compound **1**, Figure S3: ^1^H NMR (500 MHz, acetone-*d*_6_) spectrum of compound **1**, Figure S4: ^13^C NMR (125 MHz, acetone-*d*_6_) spectrum of compound **1**, Figure S5: HSQC spectrum of compound **1**, Figure S6: ^1^H-^1^H COSY spectrum of compound **1**, Figure S7: HMBC spectrum of compound **1**. Figure S8: NOESY spectrum of compound **1**, Figure S9: ECD conformers of compound **1**, Figure S10: Microscopy images of *Alternaria alternata* treated by compounds **2**, **3**, and **7** with 128 μg/mL (I–III); the vacuolated germ tubes were circled, Figure S11: Flowchart of the isolation, Table S1: List of specimens and GenBank accession numbers of sequences used in this study.
