# Supplementary material for: Sulfoxide-Containing Bisabolane Sesquiterpenoids with Antimicrobial and Nematicidal Activities from the Marine-Derived Fungus Aspergillus sydowii LW09
Source: J Fungi (Basel). 2023 Mar 12;9(3):347. doi: 10.3390/jof9030347 (PMC10057145; doi:10.3390/jof9030347)
Supplement: Supplementary file 1 [file jof-09-00347-s001.zip › jof-2250129-supplementary.pdf]

Sulfoxide-Containing Bisabolane Sesquiterpenoids with Antimicrobial and Nematicidal Activities from the Marine-Derived Fungus *Aspergillus sydowii* LW09

Xiao Yang [1,2,†], Hongjia Yu [1,2,†], Jinwei Ren [1], Lei Cai [1], Lijian Xu [2,*] and Ling Liu [1,3,*]

[1] State Key Laboratory of Mycology, Institute of Microbiology, Chinese Academy of Sciences, Beijing 100101, China; 17633751049@163.com (X.Y.); hongjia718@163.com (H.Y.); renjw@im.ac.cn (J.R.); cail@im.ac.cn (L.C.)
[2] College of Agricultural Resource and Environment, Heilongjiang University, Harbin 150080, China
[3] University of Chinese Academy of Sciences, Beijing 100039, China
* Correspondence: xulijian@hlju.edu.cn (L.X.), liul@im.ac.cn (L.L.)
† These authors contributed equally to this work.

List of Supporting Information

Figure S1. HRESIMS spectrum of compound **1**.

Figure S2. IR spectrum of compound **1**.

Figure S3. ^1H NMR (500 MHz, acetone-d_6) spectrum of compound **1**.

Figure S4. ^{13}C NMR (125 MHz, acetone-d_6) spectrum of compound **1**.

Figure S5. HSQC spectrum of compound **1**.

Figure S6. ^1H-^1H COSY spectrum of compound **1**.

Figure S7. HMBC spectrum of compound **1**.

Figure S8. NOESY spectrum of compound **1**.

Figure S9. ECD conformers of compound **1**.

Figure S10. Microscopy images of *Alternaria alternata* treated by compounds **2**, **3**, and **7** with 128 μg/mL (I–III), the vacuolated germ tubes were circled.

Figure S11. Flowchart of the isolation.

Table S1. List of specimens and GenBank accession numbers of sequences used in this study.

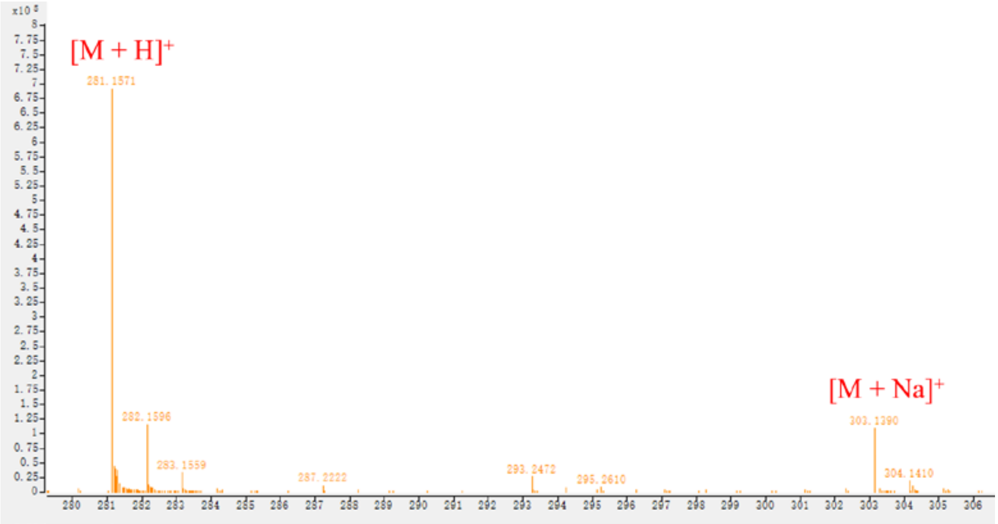

Figure S1. HRESIMS spectrum of compound **1**.

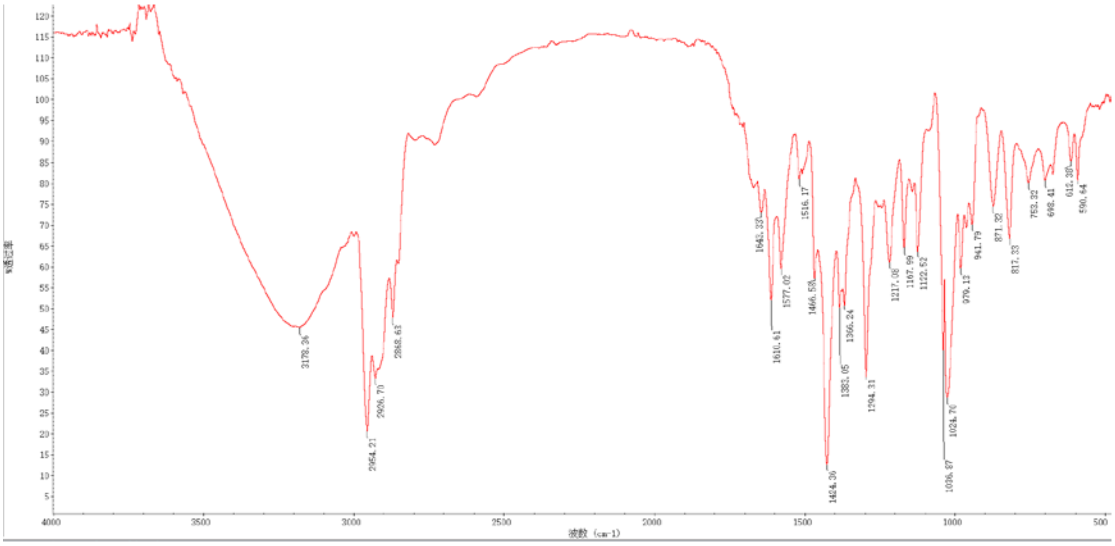

Figure S2. IR spectrum of compound **1**.

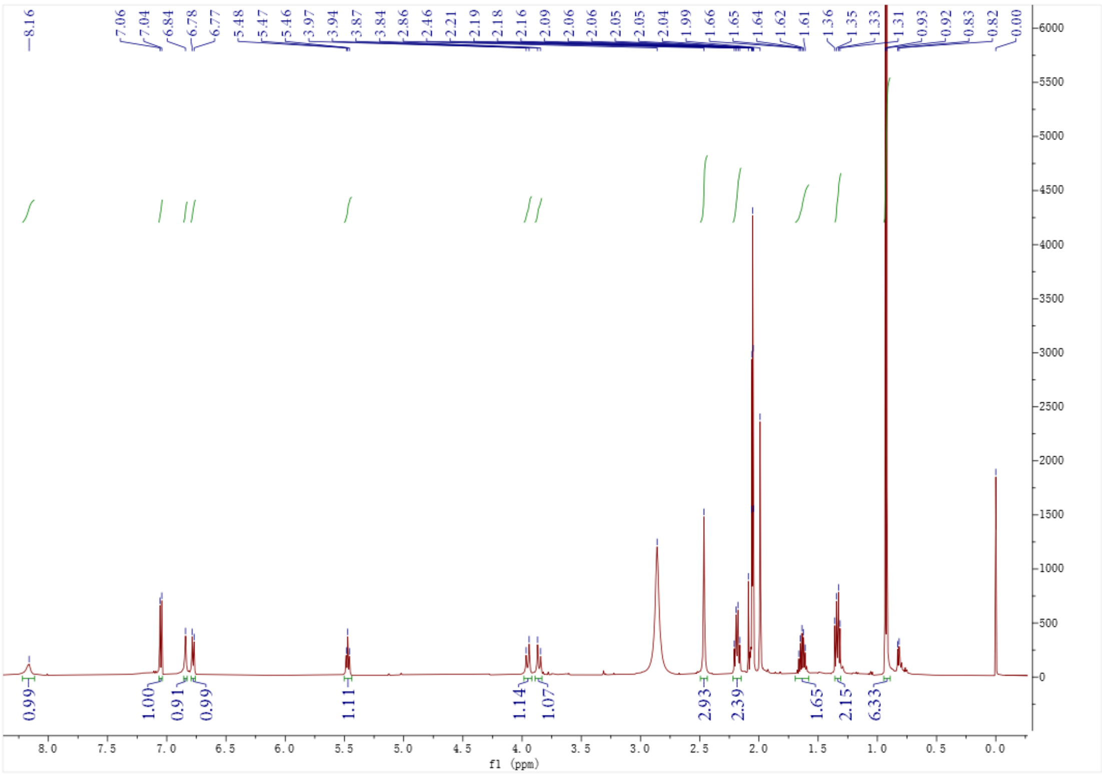

Figure S3. ^{1}H NMR (500 MHz, acetone-d_6) spectrum of compound **1**.

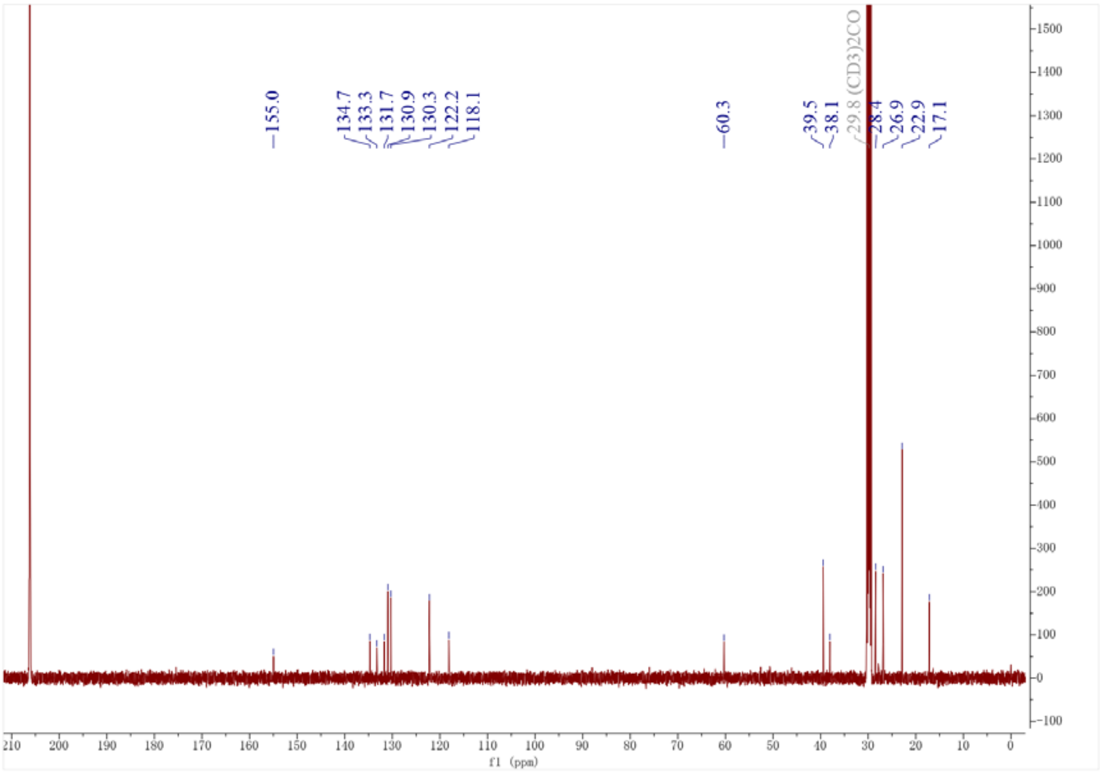

Figure S4. ^{13}C NMR (125 MHz, acetone-d_6) spectrum of compound **1**.

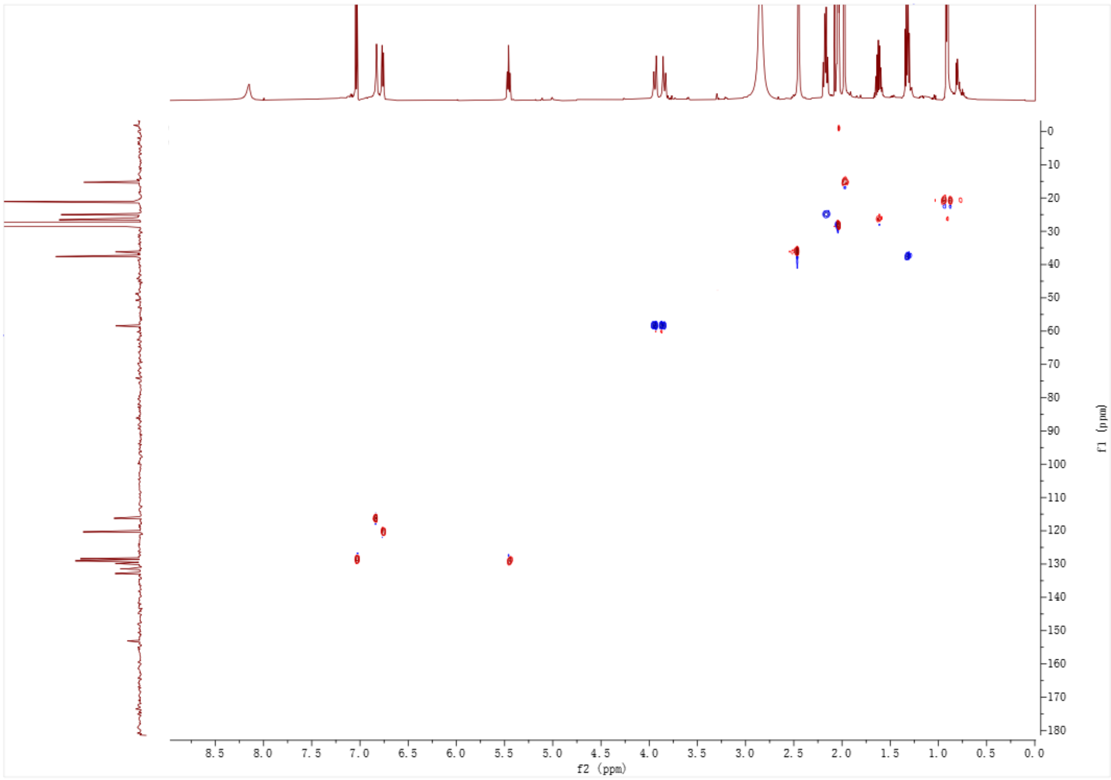

Figure S5. HSQC spectrum of compound **1**.

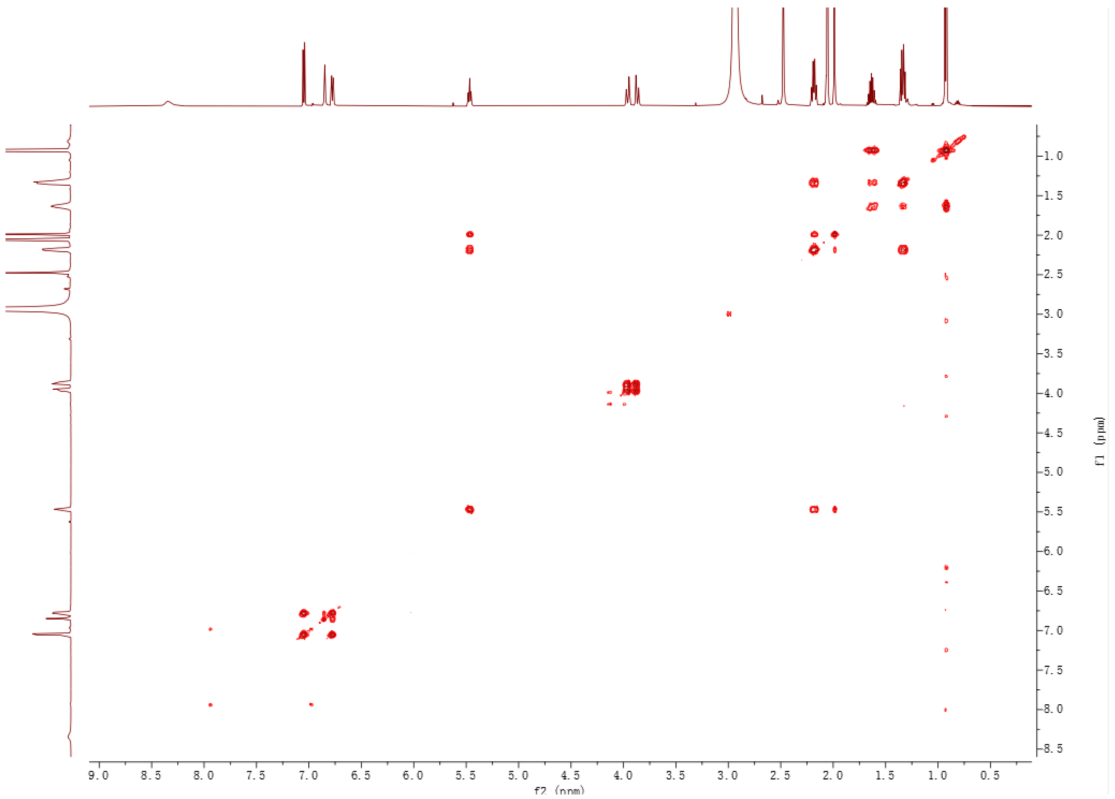

Figure S6. ^{1}H-^{1}H COSY spectrum of compound **1**.

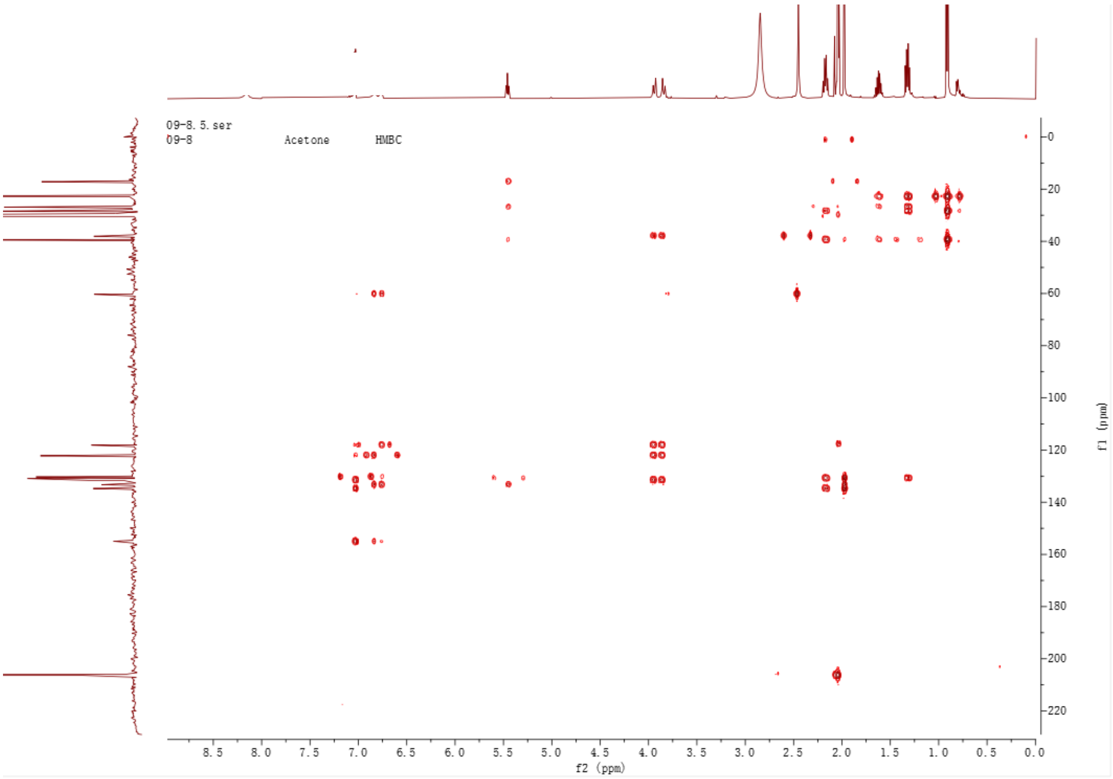

Figure S7. HMBC spectrum of compound **1**.

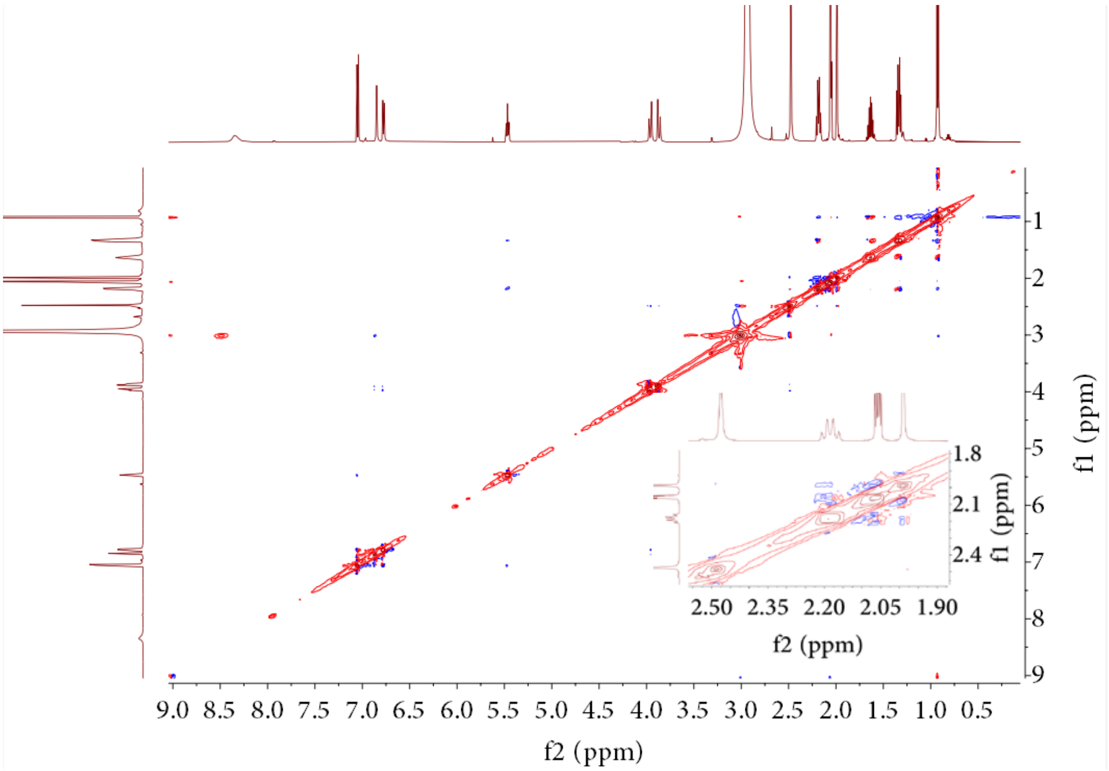

Figure S8. NOESY spectrum of compound **1**.

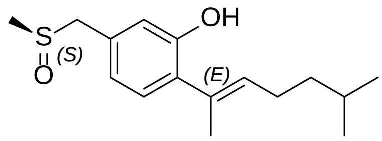	
Conformers	Populations (%)
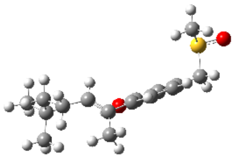	1.99
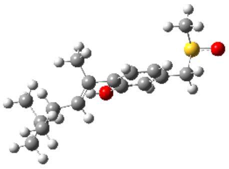	1.82
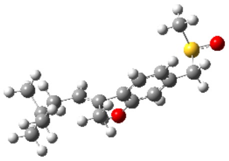	1.31
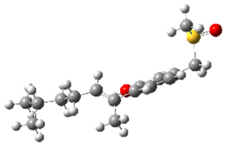	4.31

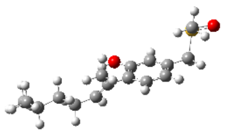	4.55
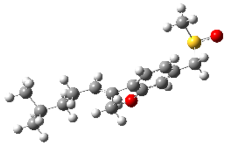	4.13
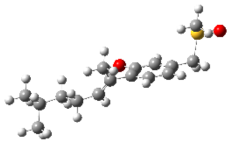	3.98
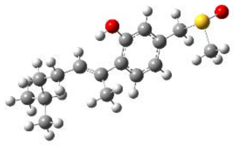	2.01
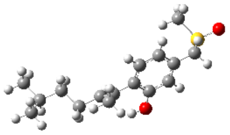	4.62

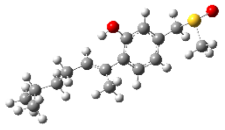	4.31
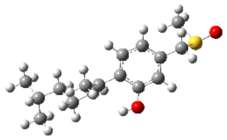	4.12
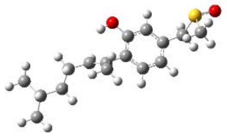	3.88
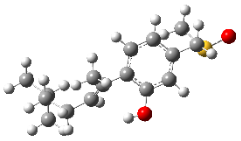	1.22
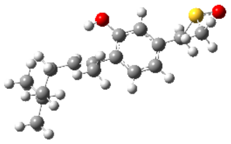	1.13

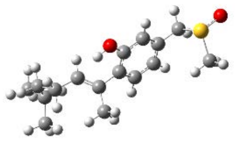	1.99
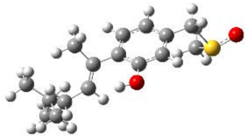	2.11
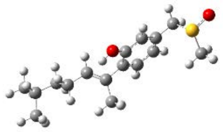	4.33
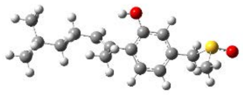	4.03
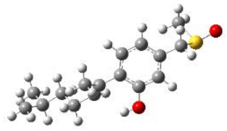	4.49

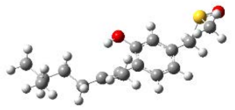	4.24
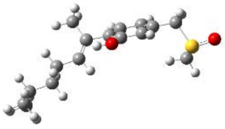	4.26
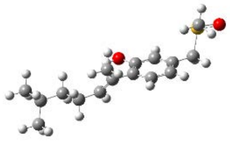	3.9
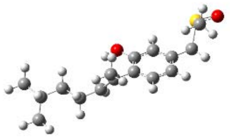	3.97
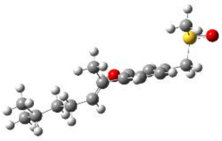	4.36

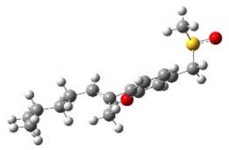	4.31
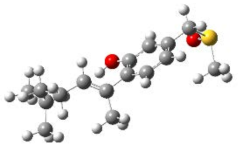	1.45
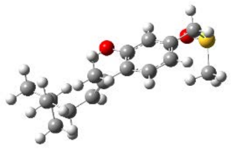	1.18
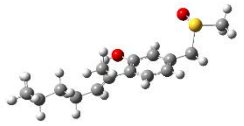	1.12
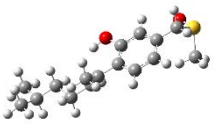	3.95

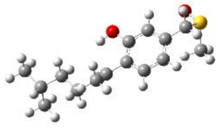	3.71
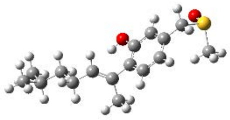	3.2

Figure S9. ECD conformers of compound **1**.

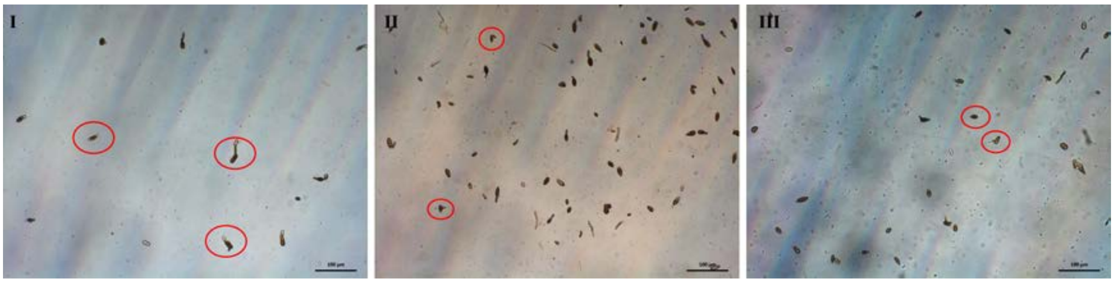

Figure S10. Microscopy images of *Alternaria alternata* treated by compounds **2**, **3** and **7** with 128 μg/mL (I–III), the vacuolated germ tubes were circled.

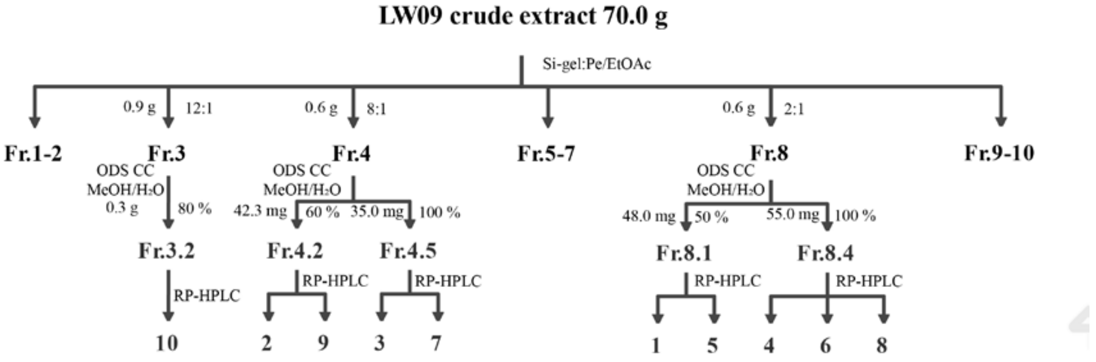

Figure S11. Flowchart of the isolation.

Table S1. List of specimens and GenBank accession numbers of sequences used in this study.

Species	Collection	ITS	BenA	CaM
Aspergillus amoenus	UTHSC 05-2980	LN898664	LN898818	LN898741
Aspergillus amoenus	UTHSC 06-1721	LN898665	LN898819	LN898742
Aspergillus amoenus	UTHSC 07-1668	LN898666	LN898820	LN898743
Aspergillus amoenus	UTHSC 08-2366	LN898669	LN898823	LN898746
Aspergillus amoenus	UTHSC 11-476	LN898670	LN898824	LN898747
Aspergillus amoenus	UTHSC 11-1419	LN898671	LN898825	LN898748
Aspergillus amoenus	UTHSC 06-4284	LN898672	LN898826	LN898749
Aspergillus amoenus	UTHSC 09-125	LN898673	LN898827	LN898750
Aspergillus amoenus	UTHSC 12-340	LN898674	LN898828	LN898751
Aspergillus amoenus	UTHSC 07-443	LN898675	LN898829	LN898752
Aspergillus amoenus	UTHSC 07-3621	LN898676	LN898830	LN898753
Aspergillus amoenus	UTHSC 09-2582	LN898677	LN898831	LN898754
Aspergillus creber	UTHSC 14-223	LN898680	LN898834	LN898757
Aspergillus creber	UTHSC 05-2359	LN898682	LN898836	LN898759
Aspergillus creber	UTHSC 09-3357	LN898684	LN898838	LN898761
Aspergillus creber	UTHSC 14-188	LN898685	LN898839	LN898762
Aspergillus creber	UTHSC 06-3435	LN898686	LN898840	LN898763
Aspergillus creber	UTHSC 10-1327	LN898687	LN898841	LN898764
Aspergillus creber	UTHSC 11-2813	LN898688	LN898842	LN898765
Aspergillus creber	UTHSC 09-2679	LN898689	LN898843	LN898766

Aspergillus creber	UTHSC 10-639	LN898690	LN898844	LN898767
Aspergillus creber	UTHSC 04-799	LN898691	LN898845	LN898768
Aspergillus creber	UTHSC 07-2788	LN898692	LN898846	LN898769
Aspergillus creber	UTHSC 04-434	LN898693	LN898847	LN898770
Aspergillus creber	UTHSC 10-582	LN898694	LN898848	LN898771
Aspergillus cvjetkovicii	UTHSC 10-479	LN898695	LN898849	LN898772
Aspergillus jensenii	UTHSC 05-3600	LN898698	LN898852	LN898775
Aspergillus jensenii	UTHSC 09-2299	LN898699	LN898853	LN898776
Aspergillus jensenii	UTHSC 10-327	LN898700	LN898854	LN898777
Aspergillus jensenii	UTHSC 12-79	LN898701	LN898855	LN898778
Aspergillus jensenii	UTHSC 07-3790	LN898702	LN898856	LN898779
Aspergillus jensenii	UTHSC 10-71	LN898703	LN898857	LN898780
Aspergillus jensenii	UTHSC 09-425	LN898704	LN898858	LN898781
Aspergillus protuberus	UTHSC 06-4104	LN898705	LN898859	LN898782
Aspergillus protuberus	UTHSC 09-246	LN898706	LN898860	LN898783
Aspergillus protuberus	UTHSC 11-269	LN898707	LN898861	LN898784
Aspergillus protuberus	UTHSC 07-2433	LN898708	LN898862	LN898785
Aspergillus protuberus	UTHSC 08-3392	LN898709	LN898863	LN898786
Aspergillus protuberus	UTHSC 11-2175	LN898710	LN898864	LN898787
Aspergillus protuberus	UTHSC 12-338	LN898711	LN898865	LN898788
Aspergillus protuberus	UTHSC 12-256	LN898712	LN898866	LN898789
Aspergillus protuberus	UTHSC 06-2837	LN898713	LN898867	LN898790

Aspergillus protuberus	UTHSC 08-1574	LN898714	LN898868	LN898791
Aspergillus puulaauensis	UTHSC 11-1436	LN898715	LN898869	LN898792
Aspergillus sydowii	UTHSC 09-48	LN898716	LN898870	LN898793
Aspergillus sydowii	UTHSC 11-204	LN898717	LN898871	LN898794
Aspergillus sydowii	UTHSC 13-2518	LN898718	LN898872	LN898795
Aspergillus sydowii	UTHSC 13-2630	LN898719	LN898873	LN898796
Aspergillus sydowii	UTHSC 06-2186	LN898720	LN898874	LN898797
Aspergillus sydowii	UTHSC 06-4167	LN898722	LN898876	LN898799
Aspergillus sydowii	UTHSC 07-1018	LN898723	LN898877	LN898800
Aspergillus sydowii	UTHSC 09-97	LN898724	LN898878	LN898801
Aspergillus sydowii	UTHSC 12-934	LN898725	LN898879	LN898802
Aspergillus sydowii	UTHSC 13-2674	LN898726	LN898880	LN898803
Aspergillus sydowii	UTHSC 10-1222	LN898727	LN898881	LN898804
Aspergillus sydowii	UTHSC 10-3180	LN898728	LN898882	LN898805
Aspergillus sydowii	UTHSC 11-2683	LN898729	LN898883	LN898806
Aspergillus sydowii	UTHSC 06-727	LN898730	LN898884	LN898807
Aspergillus sydowii	UTHSC 08-3215	LN898731	LN898885	LN898808
Aspergillus sydowii	UTHSC 09-1708	LN898732	LN898886	LN898809
Aspergillus sydowii	UTHSC 12-3109	LN898733	LN898887	LN898810
Aspergillus sydowii	UTHSC 08-865	LN898734	LN898888	LN898811
Aspergillus sydowii	FMR 14440	LN898735	LN898889	LN898812
Aspergillus tabacinus	UTHSC 03-1197	LN898736	LN898890	LN898813
Aspergillus tabacinus	UTHSC 07-2427	LN898737	LN898891	LN898814

Aspergillus tabacinus	UTHSC 10-1677	LN898738	LN898892	LN898815
Aspergillus tabacinus	UTHSC 08-2898	LN898739	LN898893	LN898816
Aspergillus versicolor	UTHSC 03-3679	LN898740	LN898894	LN898817
Aspergillus aurantiobrunneus	NRRL 4545	EF652465.1	EF652289.1	EF652377.1
